# The pathogenicity and virulence of Sindbis virus

**DOI:** 10.1080/21505594.2025.2609389

**Published:** 2025-12-24

**Authors:** Kevin J. Sokoloski, Deepa Karki, Cierra M. Isom, Sayra Moni

**Affiliations:** a Department of Microbiology and Immunology, University of Louisville, Louisville, KY, USA; b Center for Predictive Medicine for Biodefense and Emerging Infectious Diseases, University of Louisville, Louisville, KY, USA

**Keywords:** Sindbis virus, viral arthritis, alphavirus, arbovirus, neurovirulence, immune evasion

## Abstract

Sindbis virus (SINV), a widely distributed alphavirus, is both a foundational model for viral replication studies and an underrecognized human pathogen. Despite its typically mild presentation, SINV can lead to prolonged joint pain and, in rare cases, neurological complications. This review explores SINV’s molecular biology and clinical manifestations, particularly its role in causing Sindbis Fever – a self-limiting but potentially chronic arthritic disease. Molecular insights reveal mechanisms of immune evasion, neurovirulence, and persistent infection, highlighting SINV’s potential for broader public health impact, especially under changing climatic conditions. This review also identifies key virulence determinants and discusses the virus’s utility as a model for studying alphaviral encephalitis. Continued research is essential to better understand SINV pathogenesis and to prepare for potential outbreaks.

## Introduction

Sindbis virus (SINV) is an enveloped single-stranded positive-sense RNA virus belonging to the family Togaviridae [1,2]. Like many other members of the genus Alphavirus, SINV is maintained during the enzootic cycle via cyclic transmission between avian vertebrate hosts and vector competent mosquitoes. Epizootic spill-over occurs when SINV-infected mosquitoes feed upon humans or equids leading to their infection. Unlike the avian hosts of the enzootic cycle, both humans and equids are presumed to be dead-end hosts regarding transmission, as SINV fails to reach a high viremia precluding the infection of the blood-feeding mosquito [3–5]. In humans, SINV is the causative agent of an infectious musculoskeletal disease known as Sindbis Fever, which is also referred to as Pogosta Disease in Finland [6], Ockelbo Disease in Sweden [7,8], and Karelian Fever in Russia [6,9]. SINV is best known as the model or archetypic species of the genus alphavirus due to the extensive use of SINV in the pioneering/foundational work examining alphaviral molecular biology and replication [1,2]. On the basis of relative clinical severity to other alphaviruses, SINV is often overlooked as a pathogen. Nevertheless, SINV remains a human pathogen of significant concern due to its capacity to cause severe disease in otherwise healthy individuals during regular seasonal outbreaks in endemic areas and large-scale regional outbreaks [3,7,9–32].

SINV was first isolated from *Culex* mosquitoes in the community of Sindbis in the Nile River delta near Cairo, Egypt in 1952 [33]. The first human cases of SINV Fever were described nearly a decade later in Central and Southern Africa, notably in Uganda in 1961 and later in South Africa in 1963 [10,11]. It was during these outbreaks of mosquito-borne febrile illness that SINV was identified as a causative agent of an infectious rash-arthritis syndrome.

Phylogenetic analyses of SINV and SINV-like viruses led to the establishment of at least six genotypes which correlate spatially with bird migratory patterns [34], with Sindbis virus strain AR339 being the type species of Genotype I (referred hereto forth as G1). More recently, the viruses belonging to G5 have been demarcated as a separate species from SINV, with Whataroa virus (WHAV) as the type species [35,36]. Indeed, despite similarities across the remaining SINV genotypes, mounting evidence strongly supports further demarcation of the other genotypes as distinct viral species from those of G1. The species demarcation criteria for the Togavirus family set forth by the International Committee on the Taxonomy of Viruses (ICTV) currently includes i) nucleotide and amino acid sequence divergence, ii) antigenic characteristics, iii) vector association, iv) host association, v) disease association (pathogenesis), and vi) ecological characteristics [36]. Generally, a 10% divergence in amino acid sequence is considered an indicator of potential speciation, but the conservative demarcation of new species requires a combination of demarcating factors due to some members of the family exhibiting a high degree of sequence variation, such as that observed with VEEV [36].

Recent efforts examining SINV genotype diversity have utilized comparative phylogenetics to examine the relationships and distances between the SIVN genotypes and provided robust rationale for the demarcation of the SINV genotypes as unique species [37]. Considering the sequence diversity thresholds set forth by the ICTV, nearly all of the SINV genotypes meet the criteria for demarcation as G2, G3, and G6 all differ from G1 by at least 23% nucleotide divergence and 10.28% amino acid divergence [37]. Sequence comparisons also strongly infer that the G2 and G3 genotypes are highly interrelated and should be unified under a single genotype, with Argyle virus (ARGV) as the representative member (as proposed by Michie et al. [37]. Notably, only G4 fails to meet the conservative amino acid demarcation requirement relative to G1, as the range of amino acid divergence is 7.40 to 8.89 percent [37].

Beyond sequence identity, divergent antigenic characteristics is a property by which a need for demarcation may be indicated. Comparative analyses of sera from G1 and G5 infections revealed antigenic distinction between the two genotypes, which together with amino acid divergence, was used as evidence supporting the demarcation of WHAV (G5) as a distinct alphavirus species [38]. Similarly, a study examining the capacity of anti-AR339 (G1) monoclonal antibodies to recognize the viral glycoproteins of G2 and G3 viruses revealed antigenic distinctiveness amongst the three genotypes in question [39]. Coupled with the sequence divergence noted in the paragraph above, these antigenic differences support the demarcation of G2 and G3 as a unified unique species, with ARGV as the type species [37]. Members of G4, namely Kyzylagach virus and XJ-160 viruses, have shown antigenic similarities to G1 members in that neutralizing G1 antibodies can reduce the infectivity of G4 viruses; however, the effects are not reciprocal, as in antibodies neutralizing G4 viruses poorly neutralize G1 viruses, indicating that there is antigenic divergence between the two genotypes [40,41].

The use of vectorial and host association as demarcating criteria by which the individual SINV genotypes may be deemed unique species is difficult to effectively parse. This is primarily due to the members of G1 (and all SINV-like genotypes) exhibiting broad host tropism. Thus, in the absence of exhaustive comparative discriminative analyses of vector competence and host range for each of the SINV-like genotypes in a controlled laboratory setting, it is difficult to build arguments for or against demarcation based on vector and host association. Despite this complexity there are notable instances where arguments for demarcation may be made based on reservoir host species, such as the observation that herons are the probable reservoir host for G4 viruses [42], whereas grouse and Passeriformes are implicated for G1 viruses in Northern Europe [17,43–45].

Disease association is a more straightforward criterion that supports the demarcation of G1 from all other genotypes, as only members of G1 are associated with human disease [4,18,21,25,26,46]. While serological evidence supports the presence of human infections for other genotypes, no clinical illnesses have been reported in areas with endemic non-G1 genotypes [35,41]. Hence, one of the conditions set forth by the ICTV for demarcation is universally achieved for all SINV-like genotypes.

Finally, ecological characteristics serve to both separate and confound the demarcation of the individual genotypes. Due to migratory patterns of the probable avian hosts many of the genotypes exhibit broad regional distribution which may overlap, with G1 common to Europe, Africa, and the Middle East; G2 and G6 from Australia; G3 from Southeast Asia; G4 from Asia and the Middle East; and G5 (WHAV) from New Zealand [34]. Nonetheless, in specific instances ecological characteristics may be used as an effective demarcation criterion, as G6 is highly geographically restricted to the south-west region of Western Australia, leading Michie et al. 2023, to promote G6 as a distinct species with Thomson’s Lake virus (THLV) as the type species [37].

**Figure 1.**
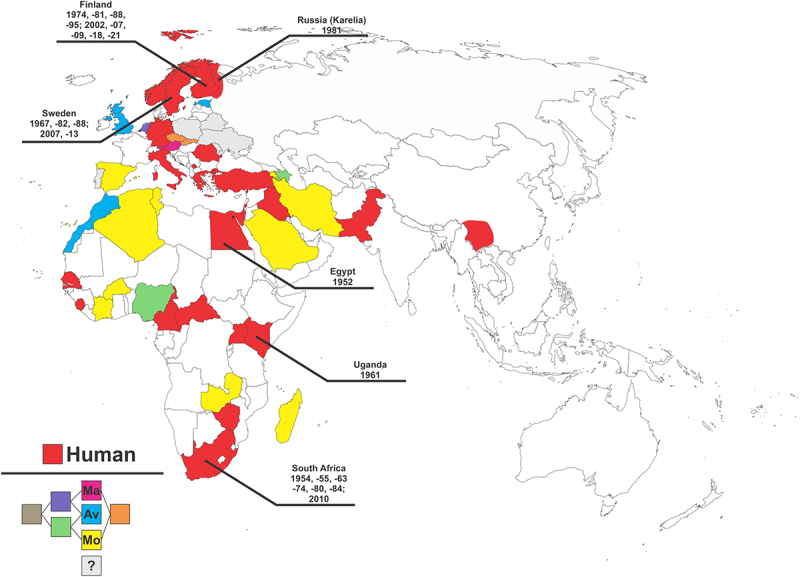
Sindbis virus is a widely distributed arthropod-borne pathogen.

Altogether, the satisfaction of multiple demarcation criteria for G2/3 (ARGV) and G6 (THLV) indicates that they are not simply SINV-like viruses, but rather that they are unique species distinct from G1 (SINV), as was previously established for G5 (WHAV). While the overall precise nature of the relationship between G1 and G4 is less defined, we posit that there is sufficient evidence to propose that demarcation is warranted based on borderline sequence identity, antigenic distinction, probable differences in avian reservoir hosts, and pathogenic differences. Accordingly, as there is sufficient evidence supporting complete demarcation, this review will focus on G1 viruses, or Sindbis virus proper, with AR339 as the representative type strain. Shortly after the discovery of SINV human cases were also reported in Northern Europe in 1967 [14–16,20], and more recently, SINV has been reported to have been detected in mosquito populations in southwestern Spain [32], suggesting ongoing expansion of the geographic range. As shown in Figure 1, and further detailed in Supplemental Data Table 1, SINV exhibits a wide geographic range with infections reported across the European and African continents and, and the Middle East [35]. The geographic range of SINV is predominantly a function of the presence and relative abundance of vector competent mosquito species, typically *Culex spp*., and suitable avian reservoir hosts, such as grouse and passeriformes [13,26,45,47,48]. Accordingly, it is unsurprising that the global distribution of SINV follows migratory routes, and endemic regions are commonly associated with wetland environments, humid forestlands, or settings where stagnant water is likely to form during warm summers [13,17,44,45,47,49–52].

A map identifying specific countries reporting SINV infections of Humans (Red), Mammals (Magenta), Avians (Blue), and Mosquitoes (Yellow). Countries where SINV has been detected in humans may be reasonably assumed to also have infections, recognized or unrecognized, in avian and mosquito populations. Countries where SINV has been detected in one or more of the nonhuman categories listed above may be identified by their color combination, as defined in the lower left corner of the figure. Countries with a gray coloration have had reports of SINV detection but the literature is unclear as to the precise host species. The entire country is highlighted even if SINV is regionally limited, with the exception of Russia as SINV has only been detected in the region of Karelia, and China where SINV (genotype 1) has only been reported in Yunan Province. Significant outbreaks of SINV in human populations are indicated by black text indicating the country affected and year of occurrence. North and South America are not included in the figure as there have been no reports of detection in either animals or humans despite widespread distribution of *Culex spp*. mosquitoes. Specific references for the map may be found detailed in Supplemental Data Table 1.

Perplexingly, local transmission of SINV (or a SINV-like virus), has not been detected on the North or South American continents despite all required environmental factors being present. The fact that Western Equine Encephalitis (WEEV) is a recombinant of Eastern Equine Encephalitis virus (EEEV) and SINV suggests that SINV was at one time present (estimated to be approximately 1300 to 1900 years ago) in close proximity to EEEV (which is exclusive to the American continents) [53].

Despite a widespread geographic range, most reported human cases are from Northern Europe where the virus is endemic, and large outbreaks occur intermittently at an almost cyclical rate of several years [18,47]. This increased burden may also be due to generalized SINV-awareness, as notably, SINV is a reportable/notifiable disease in Finland, where active surveillance systems are in place [54,55]. It is from outbreaks in these regions that the ecology and epidemiology of SINV is best understood. Due to this fact, and the often-mild self-limiting nature of infection, it is difficult to accurately estimate the annual burden of SINV disease on a global basis. In endemic areas general seroprevalence rates range from 3 to 10%, with the likelihood of seroconversion increasing with age [23,55–57]. During SINV outbreaks the observed incidence rates vary but may achieve a notably high rate for a mosquito-borne viral infection, as illustrated by the 2021 outbreak in Finland where peak incidence rates were in excess of 40 cases per 100k population [54]. Factors driving SINV outbreaks largely include those that impact the vector mosquito populations such as rainfall, temperature, and other climate-related phenomena, which have been demonstrated to impact a number of mosquito-borne pathogens [58–60].

## Sindbis Fever, Sindbis virus as a human pathogen

As an arbovirus, SINV infection and disease begins with an infected mosquito depositing viral particles into the vertebrate host through the injection of virus-containing mosquito saliva while feeding. The viral particles, aided by the anti-inflammatory and vasodilatory nature of components of the mosquito saliva, engage with the host cells in the dermis to initiate infection [61]. In this niche the virus replicates locally in keratinocytes and dermal fibroblasts until entering the bloodstream [62,63]. The resulting viremia leads to the dissemination of the virus to other tissues in the host including muscle cells, tendons, joint cells (especially synovial fibroblasts), macrophages, and cells of the central nervous system [62–65]. The combined efforts of the innate and adaptive immune system ultimately clear the virus from the host; however, persistent infection has been reported during SINV infection in humans and mouse models of infection [66,67].

**Figure 2.**
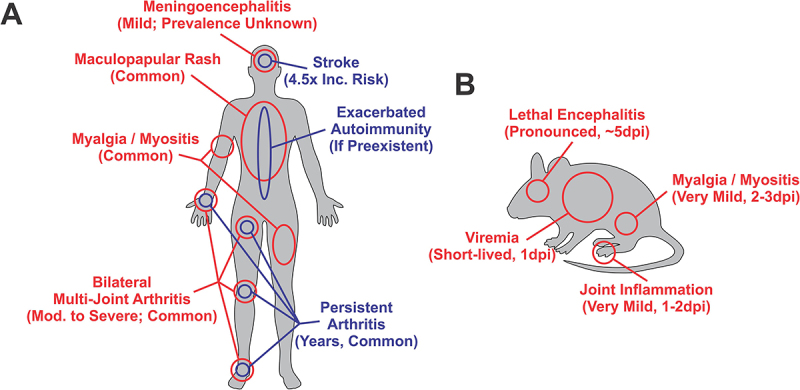
Sindbis virus disease in humans and mouse models of infection.

The ratio of subclinical to clinically diagnosed infections is estimated to range between 40 and 20:1, with underdiagnosis highly likely [21,22]. SINV infection and disease are typically thought of as self-limiting with the acute phase of infection lasting between 1 and 3 weeks; however, severe symptoms may occur in otherwise healthy individuals [4,46]. The main clinical symptoms of acute SINV infection (as depicted in Figure 2A) are mild fever accompanied by a maculopapular rash with itchy exanthema disseminated over the trunk and limbs [3,46,68,69], headache, malaise, muscle pain, and mild to severe arthritis particularly in the wrists, hips, knees and ankles [4,19,23,46]. The inflammation and joint pain associated with SINV infection appears to be exacerbated by prior joint injury or underlying arthritic conditions [70]. Most individuals suffering from clinical SINV infection recover within weeks or months, yet a substantial proportion of patients, up to 50% report experiencing chronic arthralgia for 6–8 months after infection, of which some may experience persistent joint pain and inflammation for years [66,71–73]. The persistent infection of macrophages has been observed in patients with long-term alphavirus-induced arthritis and the presence of these macrophages appears to correlate with clinical severity [74–76]. To date, despite several reports of otherwise severe disease, including a single report of recurrent hemorrhagic skin lesions [77], there have been no reported human fatalities involving SINV as the cause of death.

(A) A representation of clinical signs of SINV-induced illness in humans, with the red text representing the acute symptoms of SINV Fever. Long-term or latent impacts of SINV infection are noted in blue text. (B) For rodent models of experimental SINV infection the impacts of SINV infection are noted. For human infections the severity and commonality of occurrence is noted below in parentheses, and for mouse infections the severity and time to occurrence post-experimental infection is noted.

SINV has been investigated from a public health standpoint to determine whether or not SINV infection correlates with the later development of Rheumatoid Arthritis (RA) as the inflammatory nature of the two diseases are highly similar. Nevertheless, prior SINV infection was not found to be a risk factor for the development of RA; but, curiously, SINV infection does lead to enhanced levels of autoreactive antibodies in certain individuals particularly antinuclear antibodies, antimitochondrial antibodies, and rheumatoid factor antibodies [78]. This suggests that SINV infection may exacerbate autoimmune diseases but does not directly lead to the development of autoimmunity. In line with these observations are the findings that SINV infection does not directly lead to the development of citrulline-positive antibodies [78]. While no linkage between SINV infection and autoimmunity has been established, SINV infection, as determined by seropositivity, was associated with prior cerebral infarction or stroke by approximately 4-fold [55].

Despite SINV being ordinarily associated with musculoskeletal disease in humans, studies examining hospitalized patients with acute fever diseases of unknown causes in South Africa have indicated that SINV infection can cause meningoencephalitis and other neurological symptoms in otherwise healthy persons [79]. A similar observation was made, albeit with less frequency, during an outbreak of SINV in Northern Europe [73]. While certainly overshadowed by the neurological diseases associated with New World alphaviruses [80]; the observation that SINV infection can cause neurological disease in humans is predictable as animal models of SINV infection often demonstrate neurotropic infections. As described in more detail later, infections of other mammals including mice and horses may ultimately conclude in severe neuropathogenesis and death. Regardless, the neurological manifestations of SINV in humans tend to be mild (and commonly include confusion and weakness) and full recovery is the typical outcome [73]. The full extent to which symptomatic SINV infection contributes, and whether asymptomatic neurotropic SINV infection contributes to the later development of neuroinflammatory diseases, is unknown at this time.

## The molecular basis of Sindbis Fever in humans

Frustratingly, the pathogenesis observed during SINV infection of rodents poorly mimics the symptoms observed in humans, as arthritogenic disease is absent in mouse models of infection. While viral replication can be readily detected in joint and muscle tissues of experimentally infected mice, the overall inflammatory manifestation within the joint spaces is limited [65]. Accordingly, much of what is known, or presumed to be true, regarding alphaviral-induced arthritis stems from investigations of alphaviruses with far more robust mouse models of musculoskeletal disease such as Ross River virus (RRV) and CHIKV, or a limited number of clinical studies involving human cases of SINV Fever or primary cells taken from human donors. These generalities are described in more detail below, with specific instances where observations have been confirmed in SINV are noted.

Alphaviral-induced arthritis is the result of the immune response elicited by the production of pro-inflammatory cytokines and chemokines which promote the pathophysiological inflammatory state [65,81–83]. This response is orchestrated by cells in the synovial joints, such as the type A and type B synoviocytes (which are macrophage-like and fibroblast-like, respectively) and infiltrating immune cells responding to the infection [65,82–86]. The underlying inflammatory response induced by alphaviruses strongly resembles that observed during RA [87]; however, as noted earlier the autoimmune component is lacking [78,88]. In general, from studies using human patient data and animal models of alphaviral arthritis, acute alphaviral-induced arthritis is hallmarked by increased levels of IFNα/β, IFNγ, TNFα, IL-6, IL-1, IP-10, IL-12, IL-15 and CXCL9MCP-1 (CCL2), and MIF-I [74,83,87,89–98]. While not as exhaustively studied, SINV infections of primary human macrophages have been shown to stimulate similar cytokine expression profiles [99]. Many of the pro-inflammatory cytokines observed during the acute phase of infection are also observed in patients with chronic alphaviral-induced arthritis, in particular IL-1, IL-6 and GM-CSF and levels of these cytokines correlate with disease severity and are considered a strong prognosticator of long-term chronic arthritis [96–98]. Altogether the cytokines and chemokines produced during alphaviral infection serve to contribute to inflammation in the synovial space and eventually erosive bone damage/loss [100].

At the cellular level, macrophages which are amongst, if not the major, monocytic infiltrates into the synovial space are understood to play a central role in the development and long-term maintenance of alphaviral-induced arthritis [74,81,92,94,99]. Exposure of human macrophage tissue culture cell lines and primary human monocytes to SINV infection *in vitro* results in a robust pro-inflammatory response marked by increased levels of MIF, TNFα, IL-1, and IL-6 expression [99]. Again, akin to the pathophysiology of RA, this cytokine response leads to joint injury through the induction of matrix metalloproteinases, as MMP1 and MMP3 are strongly expressed in a MIF-dependent manner during active SINV infection [99]. The persistent infection of macrophages has been reported for both RRV and CHIKV and has been postulated as a primary driver of chronic alphavirus-induced arthritis in human and animal models of disease [74–76,92]. Whether a similar phenomenon occurs during SINV infection is unknown, but numerous similarities between the pathological etiologies indicate a strong likelihood of persistent infection.

## Sindbis as a model pathogen for alphavirus-induced encephalitis

Despite being a poor model of alphavirus-induced arthritis, SINV is an excellent model of neurotropic infection and alphaviral-induced encephalitis and has been used as a surrogate model for the encephalitic alphaviruses, namely- Eastern Equine Encephalitis virus (EEEV), Western Equine Encephalitis virus (WEEV), and Venezuelan Equine Encephalitis virus (VEEV). Despite age and mouse genotypic dependencies, the steady predictable pattern of infection and disease observed during SINV infections of mice have enabled complex dissections of the underlying pathogenic mechanisms and inflammatory responses seen during alphaviral-induced encephalitis.

Precisely why SINV infections of humans and mice results in such disparate pathological outcomes is not known and remains an area in need of further assessment. Potential mechanisms include tissue tropism differences between the hosts as determined by susceptibility and permissivity differences in the central nervous system tissues. Nonetheless, SINV has been demonstrated to infect a number of cell types found in the human central nervous system [101–103]. As such, differences in tropism are likely to be a minor contributing factor and not wholly explain the pathological differences. Alternatively, differences in the overall robustness of the host immune response to infection may limit or preclude severe neurological disease in humans. It should be noted that SINV disease in mice is largely dependent on viral strain, mouse strain, and age. Prior studies examining SINV infections of BALB/cBy and C57BL/6 mouse strains revealed the presence of resistance loci on Chromosome 2 of BALB/cBy [104,105]. This Quantitative Trait Locus (QTL), termed Nsv1, is spatially close to a number of genes involved in the innate immune response to viral infection, including IL1rn and several IL-1 cytokine family members, Psd4, and Pax8. Nonetheless, the precise mechanistic underpinnings of the Nsv1 QTL have not been fully evaluated and represents an area for future research.

## The molecular basis of SINV encephalitis in mice

Entry into the tissues of the central nervous system (CNS) is partially determined by the route of inoculation, with the subcutaneous injection model being described as most similar to the natural route of infection via mosquito bite. After the development of viremia subsequent to replication at the site of infection, SINV crosses the blood-brain-barrier (BBB) [106,107]. Precisely how this occurs has not been definitively established, but SINV is capable of infecting and replicating in endothelial cells, and the instigation of an inflammatory response is known to induce BBB permeability or result in the potential migration of infected cells into the CNS [101,108]. Entry into the central nervous system can also occur via intranasal exposure, which is presumed to bypass the BBB through the infection of neuroepithelium and neurons innervating the nasal sensory neurons and vomeronasal organ/olfactory bulb [104,109,110]. As depicted in Figure 2B, entry to the brain is relatively rapid, with viral RNA detectable as early as 1dpi, with pronounced inflammatory responses including monocyte infiltration by 3dpi [111]. Once in the brain SINV infection spreads throughout the toward the midbrain/pons and brainstem. SINV pathophysiology is readily observable via hematoxylin and eosin (H&E) staining, with common signs of alphaviral-induced encephalitis being perivascular cuffing, monocytic infiltration, gliosis, and neuronal apoptosis [103,111–115]. As infection progresses, these phenomena lead to the death of motor neurons, and eventually, ascending paralysis. Lethality is derived from the death of neurons in the brainstem [116].

After entering the CNS, SINV readily infects multiple cell types in the brain including neurons, astrocytes, and microglia. While SINV infection can lead directly to the apoptosis of infected cells, SINV neuropathogenesis is undoubtedly the result of immunopathogenesis. This is supported by the observation that SCID mice completely lacking *T*- and B-cells are resistant to SINV neuropathogenesis despite the virus replicating to high titer in the infected animals [117,118]. Infections of mouse models lacking CD4+ T cells, but not CD8+ T cells, experience reduced neuroinflammation and mortality during lethal challenge [119,120]. It was later established that T-cells, in particular CD4+ TH17 T cells, are an important contributor to the development of pathogenesis during SINV infection. After infiltration into the brain the TH17 cells contribute to pathogenesis by expressing granzyme B, IL-22, and GM-CSF leading to heightened inflammation and apoptosis [121,122]. Studies evaluating the role of T-cells in the brain during SINV-induced encephalitis have revealed that IL-10 driven responses are protective of the central nervous tissue spaces during infection, as IL-10 suppresses pro-inflammatory stimulation leading to reduced cell death during infection [121–124]. In model systems lacking IL-10 viral clearance is increased, yet the associated inflammation is exacerbated leading to greater cell death [122–124].

Numerous studies have reported the upregulation of proinflammatory cytokines and chemokines in the mouse brain following infection, with notable examples of cytokines including IFNα/β, IFNγ, TNFα, GM-CSF (CSF2), LIF, and Interleukins-1; −4; −6; −12b; −17; and −23 [109,112,118,122,124–127]. Chemokines upregulated during SINV neuropathogenesis include CCLs 2 and 5, and CXCLs 9 and 10. As the type-I IFN response is critical to limiting viral infection through the induction of Interferon Stimulated Gene (ISG) expression resulting in an antiviral state, it has been shown that nonvirulent SINV gains lethality in the absence of a functional type-I IFN response [128]. In contrast, even though IFNγ contributes to the resolution of SINV infection, IFNγ-deficient models exhibit reduced disease severity due to the alteration of the infiltrating monocyte populations in the brain [126]. Mouse models with TNFα deficiencies exhibit reduced mortality and delayed onset of paralysis indicating that TNFα is a central contributor to SINV neuropathogenesis [129,130]. Interestingly, mice deficient in IL-1β are less susceptible to SINV in lethal challenge models despite exhibiting similar patterns of apoptosis and monocytic infiltration [114].

Neuronal death during SINV infection may also occur through a means outside of what is typically considered immunopathogenesis. One such mechanism is via neuronal excitotoxicity, where neurotransmitter levels, particularly those of glutamate, accumulate in the brain leading to the overstimulation of the postsynaptic cells. Overstimulation leads to death of the neuron due to ionic disbalance and metabolic dysregulation resulting in cell death and inflammation. Glutamate toxicity is induced during SINV infection via the downregulation of GLT-1 (also known as EAAT2) which is essential for removing glutamate from the synaptic cleft. Although neurotransmitter excitotoxicity is mechanistically distinct from conventional immunopathology, the downregulation of GLT-1 is driven by TNFα and IL-1β overexpression resulting in a blurry boundary between the two mechanisms [131,132].

## SINV diagnostics and detection

In areas with endemic SINV, clinical detection is primarily based upon disease symptoms, medical history evaluations, and a general physical examination focusing on joint inflammation and pain [133]. As an arbovirus, clinical presentations of SINV disease usually follow a seasonal cycle due to the nature of the mosquito vector. People with suspected SINV infections are then evaluated using serological approaches to confirm the presence of anti-SINV IgM or IgG, typically through ELISA or other immunofluorescent clinical assays [5,134], and less often through plaque reduction neutralization assays [66]. In both methodologies, the magnitude of titer (or an increasing change in titer with time) of 4-fold is used to properly diagnose SINV infection [5]. As with all positive-sense RNA viruses, the presence of SINV is readily confirmed using RT-PCR or other nucleic acid-based detection modalities; yet, as SINV viremia concurs with the onset of symptoms and is short-lived nucleic acid-based tests are not relied upon clinically [135]. Virus isolation and culturing may also be used to confirm the presence of SINV, but the associated financial costs and specialized nature of doing so in relation to the likely clinical outcomes of SINV infection in humans precludes doing so in most cases.

## SINV treatment and prevention

Clinically, SINV Fever is treated by general supportive care and symptomatic treatment as, to date, no clinically approved specific antiviral treatments for SINV infection exists. Typical treatment regimens include antihistamines to alleviate the itchiness associated with the maculopapular rash, and nonsteroidal anti-inflammatory drugs (NSAIDs) or analgesics like acetaminophen or ibuprofen to manage joint pain [68,133]. In cases where joint pain persists, or is exceptionally severe, the intra-articular administration of corticosteroids may be employed to reduce the underlying inflammation, but there is scant support for the efficacy of this treatment and steroid treatment may exacerbate viral infections [136]. The overall similarity of SINV musculoskeletal disease and Rheumatoid arthritis (RA) has led to interest in using anti-RA treatments for alphaviral-induced arthritis; however, studies involving other alphaviruses have indicated that the use of anti-RA drugs, such as Methotrexate, may complicate alphaviral infection leading to more severe disease [137].

Like the lack of SINV-specific antivirals, unfortunately, there are no licensed or clinically approved vaccines for the prevention of SINV infection in humans or animals. While several groups have pursued research applicable to vaccines for SINV, these efforts have failed to progress as SINV acute illness is typically considered mild and rarely causes serious permanent complications.

As is common to many mosquito-borne diseases, preventative efforts on a community scale could focus on reducing mosquito populations through either vector control or denying peridomestic mosquito species access to breeding environments in close proximity to human habitats. Other physical preventative measures include wearing appropriate protective clothing, avoiding outdoors activities during the time(s) of day when mosquitoes are likely to feed (dusk and dawn), and deploying insecticide/insect repellants to reduce feeding events.

## The molecular life cycle of Sindbis virus

**Figure 3.**
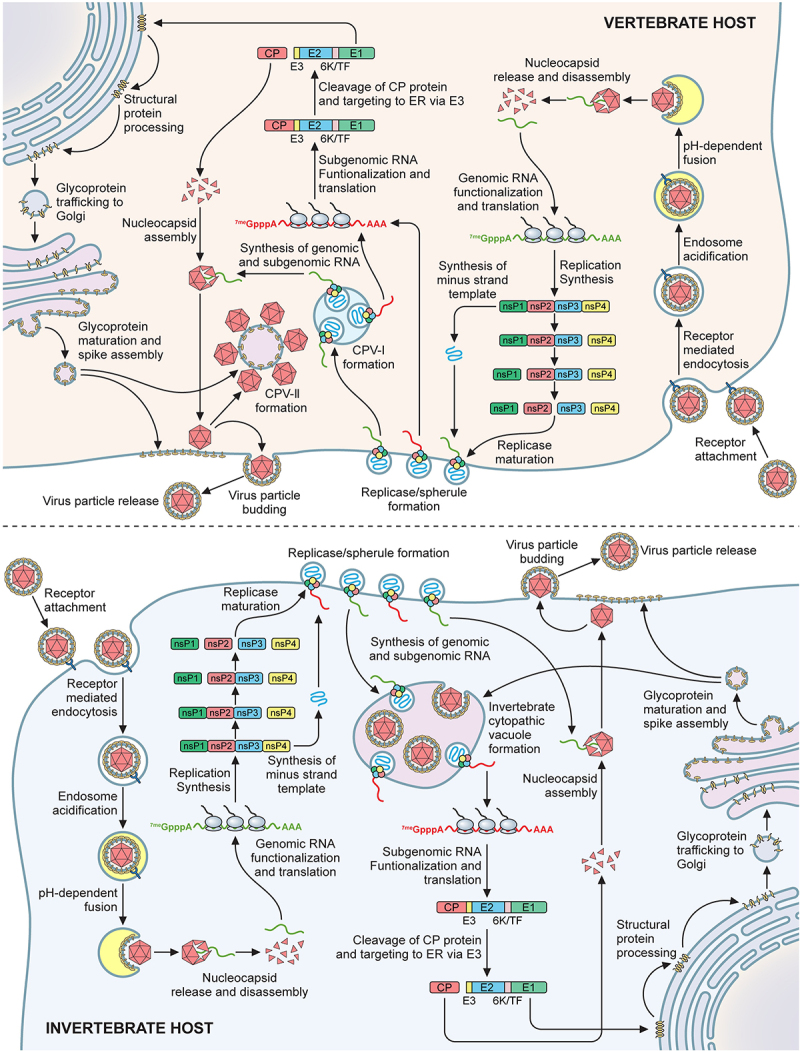
The molecular life cycle of Sindbis virus in the host cell.

As mentioned above, SINV has been used extensively as a model pathogen by which the cellular and molecular replication biology of the alphaviruses have been studied. Accordingly, much of what is known about the molecular life cycle, as diagrammed in Figure 3 was first determined for SINV and then later applied to other alphaviruses. Only more recently have other alphaviruses been extensively investigated regarding replication, with one of the most notable recent accomplishments coming from the structural characterizations of the Chikungunya virus replication complex [138]. In addition, the majority of what is understood of alphaviral molecular replication has been gleaned from studies of vertebrate model systems. Where applicable, notable differences in alphaviral replication in vertebrates and invertebrates are noted below.

A diagram detailing key events during SINV infections of susceptible and permissive host cells of vertebrate (Top; with a blue background) and invertebrate (Bottom; with a pink background) derivations. Descriptions of key events may be found in the text of the document.

At the cellular level SINV infection begins with the adsorption and attachment of SINV virions, or viral particles, to the host cell via an interaction between the viral glycoproteins and host cell attachment factors and receptors. Heparan sulfate is known to act as a ubiquitous attachment factor/co-receptor for SINV [139,140]. As SINV exhibits broad host and tissue tropism it is highly likely that SINV utilizes a multitude of host receptors, with known host receptor proteins for SINV including DC-SIGN and L-SIGN [141], NRAMP2 [142], Laminin [143], MXRA8 [144], VLDLR and ApoER2 [145]. Importantly, the underlying biology of these receptors contribute to viral entry into the host cell cytoplasm by initiating receptor-mediated endocytosis of the viral particle [146]. Maturation of the endosome results in acidification leading to the conformational rearrangement of the viral glycoproteins to expose the fusion loop of the viral E1 glycoproteins [147–150]. The burial of the fusion loops into the membrane of the endosomal vesicle initiates the membrane fusion process which culminates in the release of the nucleocapsid core into the host cell cytoplasm (as reviewed in [146]).

The SINV nucleocapsid core is made up of 240 copies of the SINV Capsid (CP) protein and one viral genomic RNA [151,152]. Previous studies have indicated that host-derived factors may also be incorporated into alphaviral nucleocapsid cores, but the specific molecular functions of these host-derived factors during the lifecycle are unclear [153,154]. The nucleocapsid core is thought to undergo a rapid disassembly process initiated by elements of the host ribosomal machinery resulting in the dispersal of the CP protein and the release of the viral genomic RNA to the host cytoplasm [148,155,156]. Shortly thereafter, the viral genomic RNA becomes functionalized and acts as a mRNA for the synthesis of the viral replication machinery via the host translational machinery and the initiation of viral RNA synthesis/replication.

The SINV genomic RNA is approximately 12kb in length and has a 5’ type-0 ^7me^GpppA cap structure and a 3’ polyadenylated tail [157]. In a manner identical to cellular mRNAs, the SINV genomic RNAs consist of protein-coding sequences flanked by 5’ and 3’ Untranslated Regions (UTRs) which serve to functionalize the viral RNA during infection. The sequence of the genomic RNA contains two coding-regions; however, only the 5’ open reading frame (ORF) corresponding to the viral replication machinery is translated from the genomic RNA. Thus, in the context of the genomic RNA the 3’ coding region exists as an extended 3’UTR unique to the viral genomic RNA.

Translation of the genomic RNA results in the synthesis of a pair of high-molecular weight polyproteins named P123 and P1234, depending on whether readthrough of the Opal stop codon between the nsP3 and nsP4 coding regions occurs [158,159]. In SINV, the Opal stop codon is part of a type II programmed ribosomal readthrough motif which leads to the synthesis of P1234 at a relative rate of ~ 5 to 20% in a temperature and host-dependent fashion [160]. These polyproteins are subsequently processed in a stepwise manner via the activity of the nsP2 protease domain in cis- and trans- to form functional replication complexes [161]. Initially, the transcriptionally inactive P1234 polyprotein is processed to liberate nsP4 to form the P123+nsP4 replication complex which predominantly synthesizes full length negative-sense viral RNAs colloquially referred to as the minus strand RNA [160]; however, the P123+nsP4 replication complex can also produce full-length positive-sense genomic RNAs [162]. As the translation of the viral genomic RNA continues resulting in the accumulation of viral nonstructural proteins, the half-life of the P123+nsP4 complex diminishes as the P123 polyprotein is rapidly processed to form nsP1 and a P23 cleavage intermediate [160,163–166]. Importantly, the cleavage of the nsP1 protein from the polyprotein marks the cessation of the synthesis of the minus strand template RNAs [160,167]. While the nsP1+P23+nsP4 replication complex is fully capable of synthesizing both the positive-sense genomic and subgenomic viral RNAs, the P23 intermediate is exceptionally short-lived due to increasing nonstructural protein accumulation [168]. As such, the synthesis of positive-sense genomic and subgenomic viral RNAs (which are produced at an excess rate of approximately 5 subgenomic RNAs per genomic RNA), is primarily the function of the fully mature replication complex consisting of nsP1, nsP2, nsP3, and nsP4 [160,167]. In contrast to the genomic RNA, the subgenomic RNA consists of solely the structural ORF accompanied by a unique 5’ UTR and a 3’UTR sequence of approximately 250 bases that is common to the genomic RNA.

The specific roles and molecular functions of the alphaviral nsPs during infection have been the subject of numerous thorough reviews [169–171]. Accordingly, the specificities of the nsPs outside of their roles as virulence factors will not be a subject of review here.

In vertebrates, the progression of the processing of the viral replicase complex is synchronized during infection due to the proteolytic processing of the polyprotein and the shutoff of host transcription via the activity of nsP2 protein, and translation via an unknown component of the subgenomic RNA/in response to PKR activation [172–176]. Collectively, the net result is that the synthesis of the nonstructural polyprotein wanes as the viral replicase complex becomes fully mature. The shutoff of host macromolecular synthesis is cytotoxic, and highly permissive host cells undergo cell death because of infection. In contrast, host transcription and translation are not significantly perturbed in invertebrate host cells resulting in the ongoing synthesis of nonstructural polyprotein throughout SINV infection [177].

As is true for most positive-sense RNA viruses, SINV replication is associated with host membranes [149,178,179]. These membranous compartments are referred to as spherules [149], and via recent investigations of the CHIKV replication complex using cryo-Electron Tomography (cryo-ET) have revealed a dodecameric ring of nsP1 proteins at the neck of the spherule which serves to stabilize the pore in addition to positioning the remaining nsP2 and nsP4 components in the neck of the ring-like structure [138]. The positioning of nsP3 is less clear but is believed to be outside the spherule in close association with the nsP2 protein. The negative-sense template minus strand RNA in duplex with the nascently synthesized positive-sense viral RNA is housed within the cocoa-pod like structure of the spherule, with the positive-sense viral RNA ultimately being extruded through the pore into the cytoplasm [180]. In vertebrate systems the replication spherules form on the plasma membrane where extensive arrays of the replication spherules form during viral replication, and large vacuolar structures deemed Cytopathic Vacuole type-I (CPV-I) are formed via endocytic pathways [181–184]. Formation of CPV-I structures is not required for viral RNA replication; however, the formation of CPV-I structures requires spherule formation, which is in turn dependent on the presence of a full complement of viral replication machinery and viral RNA synthesis [178,180,185–187]. The extent to which CPV-I structures are formed during infection varies across the alphaviral species, with evidence for SINV being on the lower end relative to other alphaviruses such as Semliki Forest virus (SFV) [178]. For SFV these cytoplasmic membranous structures have been determined to be derived from endosomal/lysosomal membranes and are dependent on host processes, including the activation of the PI3K-Akt-mTOR pathway [188–190]. Several hypotheses for the mechanistic importance of the CPV-I structures have been proposed, including the evasion of the induction of cellular responses via the obscuring of the viral replication intermediates [190], and a hand-in-hand role in assembly during nucleocapsid formation [184].

During infections of mosquito cells, vacuolar structures akin to those formed during vertebrate infection are formed and readily identified via transmission electron micrography. Regardless, these structures have notable differences when compared to the CPV-I structures, the primary of which being the presence of intact internally budded viral particles within the vesicle lumen. Additionally, the replication spherules are derived from internal endocytic membranes rather than the plasma membrane [184].

Translation of the subgenomic RNA leads to the production of the viral structural polyprotein, which, after proteolytic processing via viral and host proteases, yields the viral CP protein, E3; E2; and E1 glycoproteins, and either the 6k or TF protein [191]. The ordering of the SINV structural proteins encoded by the structural ORF is CP-E3-E2-6k/TF-E1 and depending on whether a programmed frameshift occurs, the polyproteins resulting from translation of the subgenomic viral RNA are either CP-E3-E2-6K-E1 or CP-E3-E2-TF as the frameshift that results in the production of TF abrogates the synthesis of the E1 glycoprotein. In all cases, the CP protein autoproteolytically cleaves itself from E3 during translation [151,192]. The recognition of a signal sequence by the host translational machinery in the N-terminus of E3 directs the remaining structural polyprotein synthesis to occur via the host endoplasmic reticulum (ER) where host peptidases are responsible for cleaving the structural polyprotein into the individual structural proteins [193–195]. As with the nonstructural polyprotein, the cleavage of the structural polyproteins occurs in a stepwise fashion. Initially, the CP-E3-E2-6K-E1 polyprotein is cleaved at the E2-6k/TF and 6k-E1 junctions by host Signalase to form the precursor protein PE2, which consists of the E3-E2 glycoproteins, and the E1 glycoprotein, respectively [196]. In the context of the PE2 protein, the E3 protein serves to orchestrate the structural maturation of the E2 protein until its later cleavage into the mature E3 and E2 monomers via the host furin protease [197]. The PE2 and E1 glycoproteins form a complex that continues to be proteolytically processed and structurally matured and glycosylated in the Golgi apparatus, eventually resulting in their trafficking to the plasma membrane as trimers of E1 and E2 heterodimers [198]. While some alphaviruses retain the E3 protein as part of their viral particle, SINV does not include E3 as part of the mature final virion [199]. The synthesis of the CP-E3-E2-TF polyprotein occurs at a rate of ~10–15% [200,201], and is similarly processed to that described above. Nevertheless, as the programmed frameshift abrogates the production of E1 it is unclear as to how the PE2 formed from this polyprotein ultimately contributes to the formation of the mature glycoprotein spike complex. Although similar at the N-terminus, and both being palmitoylated via host machinery, the 6k and TF proteins have diverging functions during the viral lifecycle. The 6k protein has been ascribed viroporin activity and has been shown to facilitate CPV-II formation and viral assembly despite not being incorporated into viral particles [202–204]. The TF protein is incorporated into mature viral particles and is also involved in viral particle assembly as mutants lacking TF form irregular viral particles with structural abnormalities [205–208].

The final stages of the SINV lifecycle involve the assembly of nascent viral particles from the accumulating viral RNAs and structural proteins. Much of the alphaviral assembly process remains unclear, but the formation of specific subassembly intermediates leading to formation of mature viral particles is clear [209]. One such subassembly intermediate is the formation of new cytoplasmic nucleocapsid cores once the viral CP protein recognizes the packaging signal (PS) present in the viral genomic RNA [210–212]. The precise location of the PS differs amongst the alphaviruses, where in SINV the PS is present in the nsP1 gene [213]. Regardless, the recognition of PS by the CP protein is essential to the specific selection of viral particle cargo and is believed to initiate the nucleation of the CP protein to the viral RNA resulting in core assembly, yet the specificities of nucleocapsid core assembly remain ambiguous [209]. The nascent nucleocapsid core then engages with the viral glycoproteins resulting in envelopment and formation of the mature viral particle [214,215]. As with the viral replication compartments formed during alphaviral infection, CPV-II’s, internal membranous structures that are rich with preassembly intermediates and fully assembled intracellular particles, are formed in vertebrate cells during assembly [216,217]. The release of viral particles from the host cell occurs at the plasma membrane following interactions of the viral glycoproteins with the cytoplasmic nucleocapsid cores or through extensive extracellular projections from the host cell [218]. Alternatively, during mosquito infections intracellular mature viral particles may be formed via budding into the membranous replication vesicle [184].

## The identification of Sindbis virus virulence traits

The knowledgebase of SINV pathogenesis with specific regards to virulence determinants is the product of many years of study using a highly advantageous approach that has been proven time and time again for many viruses. Non-neurotropic and neurotropic SINV infections are known to occur with several “wild type” strains of SINV isolated from mosquito and human populations and laboratory adapted strains. Importantly, comparative studies between these virulent and avirulent strains have led to the identification of key virulence determinants and a greater understanding of alphaviral neuropathogenesis/disease.

The prototypic strain of SINV, strain AR339 was first isolated in Egypt in 1952 by subculturing extracts from a mixed pool of *Culex spp*. mosquitoes [33]. The AR339 strain was further passaged in the laboratory resulting in the accumulation of polymorphisms that altered its phenotype, leading to the development of the infectious clone TR339, which faithfully recapitulated the mild neurovirulent properties of the original parental isolate [219–221]. Through a series of tissue culture and animal adaptation studies virulent and avirulent SINV laboratory strains were developed. Adaptation of the AR339 strain in the 1970’s led to the development of the SV1A strain which is poorly neuroinvasive but moderately neurovirulent, as well as the NSV strain which is highly neuroinvasive and neurovirulent [116,222]. In the late 1980’s a series of recombinant clones of the lab adapted HRsp (Heat Resistant small plaque) SINV isolate were developed, resulting in the pToto-derived SINV strain family, which ultimately led to a revolution in the understanding of alphaviral molecular replication [223]. As these pToto-derived strains lack the capacity to replicate *in vivo*, they do not cause SINV disease in mouse models of infection unless the mouse model is severely immunocompromised or practically neonatal [224]. Nonetheless, through recombination of the pToto, SV, and NSV strains a virulent recombinant strain TE was generated with which the role of the viral glycoproteins during pathogenesis was determined [225]. The isolation of a non-virulent SINV strain from a mosquito pool in Israel (SINV.Peleg) followed by serial passage in suckling and weanling mice led to the development of a series of adapted laboratory strains, SV (which is neither invasive or virulent), SVN (which was neurovirulent if intracranially injected), and SVNI (which was both neurovirulent and capable of neuroinvasion) [106,226]. Contemporarily with these efforts, a pair of natural SINV isolates were isolated and reported, strain AR86 and Girdwood, which were isolated from mosquito pools and a human patient, respectively [227,228]. The AR86 strain was found to be naturally neuroinvasive and neurovirulent, whereas the Girdwood strain was neuroinvasive but generally avirulent [229]. In addition, comparative analysis of virulent and avirulent strains have also led to the development of site-specific mutant strains with polymorphisms of interest in clean backgrounds such as TR339 and the Toto-derived strain family.

As mentioned above, the comparative assessments of the above viral strains have been used to define the neuroinvasion and neurovirulence determinants of SINV. The major findings of these efforts are described below in the context of either the viral life cycle or the host response to viral infection and are summarized in Table 1. Instances where a specific role or function of a viral component is integral to virus replication and hence by knock-on effect pathogenesis- for instance, the capacity of the nsP2 protease to cleave the P123 polyprotein enabling replication, are not considered separable virulence determinants and will not be discussed.

## Virulence factors affecting viral attachment and entry

The capacity for SINV to interact with host receptors is critical to the overall pathogenesis/virulence *in vivo*. Expectedly, many of the early neuroinvasion and neurovirulence determinants have been found to be within the viral E2 and E1 glycoproteins [230]. The SINV E2 glycoprotein is responsible for receptor engagement via the glycosylated ectodomain during the entry pathway whereas the endodomain of E2 extends inwards past the viral envelope where it engages with a hydrophobic pocket in the viral capsid protein [215,231–233]. The E2 protein is organized into four domains, with Domain A as the primary receptor-binding region. Domain B of the E2 protein is the apex of the glycoprotein spike complex, and Domain C mediates extensive contacts with the E1 glycoprotein and is critical for spike stability and the regulation of fusion. Domain D is internal to the viral envelope and is involved with capsid protein binding during assembly and maturation. The E1 protein is a class II fusion protein which disengages from E2 and trimerizes in response to endosome acidification during the viral entry pathway [146,234,235]. The E1 protein itself is organized into three domains. Domain I of the E1 glycoprotein is a highly conserved central domain that acts as a scaffold and is involved in the confirmational rearrangements of the spike complex during entry. Domain II, which contains the fusion loop, extends outward from Domain I and interacts with E2 to maintain the spike complex [236]. Finally, Domain III is an immunoglobulin-like fold that is attached to the stem region and C-terminal transmembrane domain and is involved in the fold-back mechanism leading to membrane fusion.

Based on the functional importance of Domains A and B of the E2 protein, it is unsurprising that a number of virulence determinants have been identified in this region. Specifically, comparative analysis of virulent and avirulent viruses led to the identification of residues R1, H55, K70, and S114 of Domain A, S114 in the Domain A-B linker, and R157 and K159 of Domain B as virulence determinants involved in the engagement of heparan sulfate (HS) moieties during the viral entry process [106,110,140,219,224,225,237–247]. In the case of the H55 residue the lysine residue at position 70 (K70) was required for full virulence [140,237]. As noted above, the residues at R157 and K159 are involved in the engagement of HS but also individually exhibited differences in replication within the cells of the central nervous system through a mechanism that is unclear [246,247]. Altogether, these mutations were ultimately found to mediate enhanced binding to Heparan Sulfate (HS), a ubiquitously expressed extracellular moiety, leading to enhanced viral attachment and entry. Along a similar vein, the mutation of E2 residues that are typically glycosylated, namely N196Q and N318Q, resulted in increased virulence presumably due to altered HS interactions [248]. In addition to the virulence determinants found in Domains A and B, several residues critical for entry/virulence have been identified in Domain C and the C-stem region of E2. These include R172, M190, S243, and K260 (which coordinates with M190), which have been shown or are postulated to be involved in receptor binding [106,229,239,249].

As part of the glycoprotein spike complex, and the source of the fusion loop, residues in the E1 protein have also been identified as major virulence determinants. These include residues A72, D75, V80; and G172, P226, and A237 of Domain II [224,225,244,250–252]; and D313 in Domain III [225]. The precise impacts of the V72A and G313D residues on E1 function are unclear, yet inferences may be made based on the location of the residues in the larger structure of the E2/E1 glycoprotein spike complex [225]. The V72A residue is close to the apical end of the E1 glycoprotein and may influence the activity of the fusion loop. The G313D residue is located in Domain III and may influence inter E1/E2 interactions during entry. Residue V80L in the E1 glycoprotein has also been demonstrated to be important for SINV infection *in vitro* in both mammalian and mosquito cell lines [250]. Likewise, residue P226S has also been shown to greatly influence SINV infection by influencing the requirement for cholesterol [252]. Other residues of E1 have been also established as important to alphaviral pathogenesis, including D75, and A237, but the precise mechanism(s) by which they act is unclear [224,225,244].

## Virulence factors affecting the host innate immune response

A robust innate immune response is critical to inhibiting viral infection and spread within the host and is key to limiting pathogenesis by reducing the overall infectious burden within the host [128]. As mentioned earlier, the effects of type-I IFN expression on alphaviral infection and pathogenesis are pronounced, as alphaviruses are susceptible to many of the IFN-stimulated effector ISGs [128]. Thus, it is unsurprising that the alphaviruses have developed a multifaceted interface with the host that includes numerous and often redundant mechanisms by which the induction of an innate immune response is evaded or blunted during an infection.

### Innate immune evasion traits of the SINV viral RNAs

Several studies noted that the primary sequence of the noncoding regions of SINV were integral to virulence. Specifically, comparative analysis of the lab adapted SVN and SVNI strains revealed that nt5 and nt8 of the SINV 5’UTR were important to neurovirulence [220,249,253]. At the time the precise reason was unclear; however, later investigations into a similar phenomenon in VEEV revealed that structures in the alphaviral genomic 5’ UTR are critical to the evasion of innate immune detection of the type-0 cap structure [254].

In addition to the virulence traits present in the SINV genomic 5’UTR, the subgenomic 5’UTR also possesses at least one element critical to pathogenesis. Downstream of the CP protein start codon, an RNA secondary structure termed the Downstream Loop element (DLP) serves to guide the initiation of translation to the proper start codon during viral infection [174,255–257]. The activity of the DLP is critical to viral structural gene expression, and by extension pathogenesis, as the host PKR pathway recognizes viral double-stranded RNA resulting in the phosphorylation of eIF2α and subsequent translational shutoff [172]. SINV replication is significantly attenuated *in vivo* in the absence of the DLP, which (although not directly shown) certainly impacts virulence [173].

Related observations have been made regarding the alphaviral 3’UTR, yet uncertainty remains regarding the presence of identified virulence traits in SINV. This is partially due to a lack of complementary investigations confirming observations that have been made in cell culture in genuine *in vivo* models of infection. Examples of this include regions of the 3’UTR known to direct either replication efficiency, translational fitness (occasionally in a host-specific manner), or RNA stability with the latter two examples being dependent on the engagement of host RNA-binding proteins [258,259]. Regardless, some known alphavirus 3’UTR virulence traits, such as the presence of miRNA binding sites in the 3’UTR of EEEV, are known to not be conserved in SINV [260]. Hence, the full impact of the 3’UTR on SINV virulence, and indeed alphaviral pathogenesis, requires further investigation.

More recently, it was demonstrated that a significant number of positive-sense RNAs lacking a 5’ cap structure are synthesized during SINV infection [261,262]. Further investigations revealed this to be common to other alphaviruses, and the production of the noncapped genomic RNA species was also observed *in vivo* [154,261]. While it is currently unclear as to precisely how these noncapped RNAs are produced during infection, the nsP1 protein, which is the viral capping enzyme [263–266], was implicated through comparative analysis of virulent and avirulent RRV strains. Taking cue from studies examining VEEV nsP1 capping activity *in vitro* [267], point mutations of the SINV nsP1 protein were demonstrated to alter their production during viral infection [262]. One such mutant, nsP1 D355A, which decreases the production of noncapped viral genomic RNAs, is significantly attenuated *in vivo* [103]. In contrast, increasing the production of the noncapped genomic RNAs via the nsP1 N376A mutation did not significantly alter neurovirulence. Paradoxically, increasing the proportion of capped genomic RNAs, despite diminishing the impact of the type-I IFN response on SINV infection, reduced the production and infectivity of SINV particles [103,262,268]. Later investigations specifically examining the impact of the noncapped genomic RNAs on viral infectivity revealed viral translational enhancement early during infection as an underlying virulence trait [268].

### Innate immune evasion traits of the SINV nonstructural proteins

Comparative studies involving the neurovirulent AR86 strain and the avirulent Girdwood strain revealed the threonine residue at position 538 as critically important to neurovirulence in the AR86 strain [269,270]. This particular residue lies within the nsP2 cleavage site between nsP1 and nsP2, and incorporating a I538T mutation in the Girdwood strain is sufficient to impart enhanced virulence, albeit modestly. The precise mechanism underlying this virulence determinant is unclear but undoubtedly involves the regulation of the type-I IFN response. Curiously, the mechanism of action of the 538T residue is independent of the host shutoff of transcription afforded by nsP2 (described in more detail below), suggesting an alternative impact on nsP1 or P123 biology [270]. Two other virulence determinants in the nsP1 protein have been described, namely T173 and R425, which are in the MTAse and Membrane Association domains, respectively [106,271]. The precise functions of these residues are unclear, but they presumably alter capping efficiency or replicase complex formation.

In addition to cleaving the nonstructural polyprotein, the SINV nsP2 protein is critical to the shutoff of host transcription through the proteolytic degradation of RPB1, the major catalytic subunit of the host RNA polymerase II complex [272,273]. Importantly, the degradation of RPB1 following SINV gene expression is the primary means by which the host response to infection is curtailed. Point mutations unable to direct the degradation of RPB1, such as the P726G mutation, are significantly attenuated and result in persistent infection at the cellular level in tissue culture models of infection [273,274]. The role of the alphaviral nsP2 protein in host transcriptional inhibition is unique to the Old-World alphaviruses, as the New World alphaviruses rely on the activity of the CP protein to accomplish a similar end [175].

The nsP3 protein, in addition to serving as a scaffold for the interaction of numerous host factors in a species-dependent manner (*both host and virus*), possesses a Macrodomain with ADP-ribosyl binding and hydrolase activities. Mutation of residues N10 and N24 are inferred to reduce ADP-ribosylase activity resulting in attenuation [275], and mutations in both the binding and hydrolase functional domains of the nsP3 protein, namely at residues 32 and 114, result in attenuation and loss of neurovirulence [276]. Mechanistically, precisely how the loss of ADP-ribosylase activity contributes to a reduction in IFN expression is unknown and in need of continued examination. The nsP3 Hyper Variable Domain (HVD) is also involved in virulence, as the deletion of residues 386 to 403 results in attenuation *in vivo*, likely through the disruption of host/pathogen interactions [229]. Potential mechanisms include the restructuring of the host stress granule microenvironment to enhance viral gene expression and replication.

While not a coding change perse, the Opal stop codon between nsP3 and nsP4 is also an important virulence trait as demonstrated by comparative studies of the virulent AR86 and avirulent Girdwood strains of SINV [229]. Specifically, SINV AR86 lacks the Opal stop codon and encodes a cysteine residue in its place resulting in the constitutive production of the nonstructural P1234 polyprotein [229]. While replacing the Opal stop codon in the AR86 strain did not eliminate virulence, mortality on the whole was reduced 2-fold and survival was prolonged by approximately 2 days ^(229)^. Precisely how the increased synthesis of P1234 enhances virulence has not been rigorously examined, yet the most likely mechanism is self-explanatory as increased synthesis of the viral replication complex leads to knock-on effects throughout the viral lifecycle (as P123 cannot replicate viral RNA in the absence of nsP4).

### Innate immune evasion traits of the SINV structural proteins

The SINV CP protein plays a critical role in the evasion of the induction of an innate immune response by masking the detection of viral Pathogen Associated Molecular Patterns (PAMPs) during infection [277,278]. Specifically, the SINV CP protein was found to interact with the host IRAK1 kinase resulting in a significantly reduced capacity to sense and respond to TLR ligands [277]. The inhibition of PAMP via TLR-based detection was found to not require viral gene expression, revealing that the viral CP protein derived from the incoming viral particles was capable of masking viral infection regardless of infectious potential [277]. Mutational analyses revealed that the inhibition of IRAK1 signaling was due to residues within the RII domain of the SINV CP protein [278]. Importantly, further studies determined that this particular function of the RII domain was highly conserved amongst the other alphaviruses. Loss of the critical interaction residues in SINV resulted in complete loss of neuroinvasiveness due to a robust innate immune response, and intracranial infections revealed altered neuropathogenesis suggesting an additional role for the interaction beyond the masking of TLR-sensing [278].

Interactions between the SINV CP protein and the viral genomic RNA have also been found to contribute to viral pathogenesis; however, the underlying mechanism remains unclear and is partially confounded by the nature of the interaction site mutants themselves [279]. The impact of the CP viral genomic RNA interaction was assessed via the disruption of the CP interaction sites by way of the incorporation of silent mutations throughout the interaction site. Of the three high specificity interaction sites, the mutation of two sites were found to be attenuating, specifically those at nt9300 and nt10100. Unfortunately, the robust attenuation observed with the nt10100 site is undoubtedly influenced by the alteration of the TF protein (which coincides with the CP RNA binding site) [206,279]. Examinations of the more mildly attenuating mutant of the nt9300 interaction site indicated a potential role in viral RNA stability and the evasion of the type-I IFN response.

As noted in the paragraph above, the TF protein, which is produced at a reduced rate to all other SINV structural proteins, is known to be important to the evasion of the host innate immune response. Indeed, mutants that produce solely the 6k protein during infection, or mutants of the TF protein which are incapable of being palmitoylated, are highly attenuated *in vivo* ^*(206)*^. Precisely how the TF contributes to the evasion of the innate immune response, directly or indirectly, is uncertain.

## Conclusions and future directions

Although SINV has been extensively used as a model alphavirus for the purposes of defining alphaviral replication biology and alphaviral-induced encephalitis, much remains unknown regarding the pathogenesis of the complex rash/arthritis syndrome known as SINV Fever in humans. SINV has been neglected as a clinically relevant arbovirus that exhibits a broad geographic range rich with immunologically naïve individuals. As time has recently demonstrated through the global outbreaks of arboviral disease, pathogens such as SINV are readily impacted by climatological changes and under the right conditions explosive outbreaks of disease may occur. As it becomes more and more apparent that prior infections and inflammatory insults may contribute to the later development of morbidities and the response to future infections, understanding pathogens that have been long presumed to be inconsequential to human health becomes increasingly important. Thus, continued SINV research is not only critical to the understanding of alphaviral infections and pathogenesis, but also to public health efforts by which future outbreaks may be mitigated.

As described earlier, a major challenge preventing a full understanding of SINV virulence is the lack of a model system that faithfully recapitulates SINV fever disease as it presents clinically in humans. To some extent the mechanisms underlying human arthritogenic alphaviral diseases, such as those of CHIKV, RRV, and MAYV, appear to be broadly applicable to human SINV infections and pathogenesis. Therefore, comparative studies across the arthritogenic alphaviruses using mouse models of infection may continue to provide meaningful insights into human SINV infections. While nonhuman primates have been used to examine the pathogenesis of other alphaviruses, such as EEEV [280], given the lack of lethal SINV infections in humans, there is little pressing need or rationale for doing so with SINV. The use of human organoids modeling the synovial joints or primary tissue samples to investigate SINV infection represents a pathway by which aspects of SINV pathogenesis may be probed in models adjacent to genuine human infections. The pairing of molecular investigations involving these model systems with clinical samples represents a means by which SINV-specific pathogenesis in humans may be better understood and the development of novel antiviral therapeutics identified.

## Supplementary Material

Sokoloski Virulence Revision Supplemental Data 1.xlsx

Supplemental Data 1 References.docx

## Data Availability

The authors confirm that the data supporting the findings of this study are available within the article, and that there is no new primary data associated with this research.
