# Supplementary material for: The pathogenicity and virulence of Sindbis virus
Source: Virulence. 2025 Dec 24;17(1):2609389. doi: 10.1080/21505594.2025.2609389 (PMC12758272; doi:10.1080/21505594.2025.2609389)
Supplement: Supplemental Data 1 References.docx [file KVIR_A_2609389_SM4340.docx]

**Supplemental Data Table 1- Geospatial Distribution of Sindbis Virus in Humans, Animals, and Insects**

**Supplemental References for Figure 1 / Supplemental Data Table 1**

**(Listed In Alphabetical Order)**

1. Avizov N, Zuckerman N, Orshan L, Shalom U, Yeger T, Vapalahti O, Israely T, Paran N, Melamed S, Mendelson E, Lustig Y. 2018. High Endemicity and Distinct Phylogenetic Characteristics of Sindbis Virus in Israel. J Infect Dis 218:1500-1506.

2. Ayhan N, Hachid A, Thirion L, Benallal KE, Pezzi L, Khardine FA, Benbetka C, Benbetka S, Harrat Z, Charrel R. 2022. Detection and Isolation of Sindbis Virus from Field Collected Mosquitoes in Timimoun, Algeria. Viruses 14. PMC9144192

3. Balducci M, Verani P, Lopes MC, Gregorig B. 1967. Survey for antibodies against arthropod-borne viruses in man and animals in Italy. II. Serologic status of human beings in a northern Italian region (Gorizia province). Am J Trop Med Hyg 16:211-5.

4. Barakat AM, Smura T, Kuivanen S, Huhtamo E, Kurkela S, Putkuri N, Hasony HJ, Al-Hello H, Vapalahti O. 2016. The Presence and Seroprevalence of Arthropod-Borne Viruses in Nasiriyah Governorate, Southern Iraq: A Cross-Sectional Study. Am J Trop Med Hyg 94:794-9. PMC4824220

5. Bergman A, Dahl E, Lundkvist Å, Hesson JC. 2020. Sindbis Virus Infection in Non-Blood-Fed Hibernating Culex pipiens Mos quitoes in Sweden. Viruses 12:1441.

6. Bergqvist J, Forsman O, Larsson P, Naslund J, Lilja T, Engdahl C, Lindstrom A, Gylfe A, Ahlm C, Evander M, Bucht G. 2015. Detection and isolation of Sindbis virus from mosquitoes captured during an outbreak in Sweden, 2013. Vector Borne Zoonotic Dis 15:133-40.

7. Blackburn NK, Foggin CM, Searle L, Smith PN. 1982. Isolation of Sindbis virus from bat organs. Cent Afr J Med 28:201.

8. Buckley A, Dawson A, Gould EA. 2006. Detection of seroconversion to West Nile virus, Usutu virus and Sindbis virus in UK sentinel chickens. Virol J 3:71. PMC1569371

9. Buckley A, Dawson A, Moss SR, Hinsley SA, Bellamy PE, Gould EA. 2003. Serological evidence of West Nile virus, Usutu virus and Sindbis virus infection of birds in the UK. J Gen Virol 84:2807-2817.

10. Darwish MA, Hoogstraal H, Roberts TJ, Ahmed IP, Omar F. 1983. A sero-epidemiological survey for certain arboviruses (Togaviridae) in Pakistan. Trans R Soc Trop Med Hyg 77:442-5.

11. De’Sean Mears C, Tabata K, Ariizumi T, Hang'ombe BM, Qiu Y, Harima H, Kajihara M, Hall WW, Sasaki M, Sawa H, Orba Y. 2025. Mosquito-borne alphaviruses in Zambia: Isolation and characterization of Eilat and Sindbis viruses. Virus Research 358:199604.

12. Drăgănescu N, Iftimovici R, Girjabu E, Iacobescu V, Buşila A, Cvaşniuc D, Tudor G, Mănăstireanu M, Lăpuşneanu. 1975. Investigations on the presence of antibodies to several alphaviruses in humans and domestic animals of a region with elevated epidemiological potential. Virologie 26:99-102.

13. Eiden M, Ziegler U, Keller M, Müller K, Granzow H, Jöst H, Schmidt-Chanasit J, Groschup MH. 2014. Isolation of sindbis virus from a hooded crow in Germany. Vector Borne Zoonotic Dis 14:220-2.

14. Ernek E, Kozuch O, Gresíková M, Nosek J, Sekeyová M. 1973. Isolation of Sindbis virus from the reed warbler (Acrocephalus scirpaceus) in Slovakia. Acta Virol 17:359-61.

15. Faye M, Ban M, Top FK, Ndiaye EH, Thiaw FD, Fall G, Diagne MM, Sall AA, Diallo M, Choumet V, Faye O. 2024. Establishment of a New Real-Time Molecular Assay for the Detection of Babanki Virus in Africa. Viruses 16. PMC11680190

16. Fourie I, Snyman J, Williams J, Ismail A, Jansen van Vuren P, Venter M. 2022. Epidemiological and Genomic Characterisation of Middelburg and Sindbis Alphaviruses Identified in Horses with Febrile and Neurological Infections, South Africa (2014-2018). Viruses 14. PMC9501102

17. Francy DB, Jaenson TG, Lundström JO, Schildt EB, Espmark A, Henriksson B, Niklasson B. 1989. Ecologic studies of mosquitoes and birds as hosts of Ockelbo virus in Sweden and isolation of Inkoo and Batai viruses from mosquitoes. Am J Trop Med Hyg 41:355-63.

18. Gaidamovich SY, Ismailov AS, Klisenko GA, Mirzoeva NM. 1978. Detection of Sindbis and West Nile viruses in the blood of living birds by indirect haemagglutination. Acta Virol 22:430.

19. Graff SL, Eibner GJ, Ochieng JR, Jones TC, Nsubuga AM, Lutwama JJ, Rwego IB, Junglen S. 2024. Detection of two alphaviruses: Middelburg virus and Sindbis virus from enzootic amplification cycles in southwestern Uganda. Front Microbiol 15:1394661. PMC11165182

20. Gresíková M, Batíková M. 1978. Comparative studies on sindbis virus strains isolated in Slovakia. Acta Virol 22:162-6.

21. Gresíková M, Sekeyová M, Tempera G, Guglielmino S, Castro A. 1978. Identification of a Sindbis virus strain isolated from Hyaloma marginatum ticks in Sicily. Acta Virol 22:231-2.

22. Gresiková M, Thiel W, Batiková M, Stünzner D, Sekeyová M, Sixl W. 1973. [Haemagglutination-inhibiting antibodies against arboviruses in human sera from different regions in Steiermark (Austria). I (author's transl)]. Zentralbl Bakteriol Orig A 224:298-302.

23. Guarido MM, Fourie I, Meno K, Mendes A, Riddin MA, MacIntyre C, Manyana S, Johnson T, Schrama M, Gorsich EE, Brooke BD, Almeida APG, Venter M. 2023. Alphaviruses Detected in Mosquitoes in the North-Eastern Regions of South Africa, 2014 to 2018. Viruses 15. PMC9965626

24. Gutiérrez-López R, Ruiz-López MJ, Ledesma J, Magallanes S, Nieto C, Ruiz S, Sanchez-Peña C, Ameyugo U, Camacho J, Varona S, Cuesta I, Jado-García I, Sanchez-Seco MP, Figuerola J, Vázquez A. 2025. First isolation of the Sindbis virus in mosquitoes from southwestern Spain reveals a new recent introduction from Africa. One Health 20:100947. PMC11699435

25. Hanafi-Bojd AA, Motazakker M, Vatandoost H, Dabiri F, Chavshin AR. 2021. Sindbis virus infection of mosquito species in the wetlands of northwe stern Iran and modeling the probable ecological niches of SINV vectors in the country. Acta Tropica 220:105952.

26. Hesson JC, Lundström JO, Tok A, Östman Ö, Lundkvist Å. 2016. Temporal Variation in Sindbis Virus Antibody Prevalence in Bird Hosts in an Endemic Area in Sweden. PLOS ONE 11:e0162005.

27. Hesson JC, Verner-Carlsson J, Larsson A, Ahmed R, Lundkvist Å, Lundström JO. 2015. Culex torrentium Mosquito Role as Major Enzootic Vector Defined by Rate of Sindbis Virus Infection, Sweden, 2009. Emerg Infect Dis 21:875-8. PMC4412225

28. Hubálek Z. 2008. Mosquito-borne viruses in Europe. Parasitol Res 103 Suppl 1:S29-43.

29. Jalava K, Sane J, Ollgren J, Ruuhela R, Rätti O, Kurkela S, Helle P, Hartonen S, Pirinen P, Vapalahti O, Kuusi M. 2013. Climatic, ecological and socioeconomic factors as predictors of Sindbis virus infections in Finland. Epidemiol Infect 141:1857-66. PMC9155282

30. Jöst H, Bialonski A, Storch V, Günther S, Becker N, Schmidt-Chanasit J. 2010. Isolation and phylogenetic analysis of Sindbis viruses from mosquitoes in Germany. J Clin Microbiol 48:1900-3. PMC2863933

31. Jöst H, Bürck-Kammerer S, Hütter G, Lattwein E, Lederer S, Litzba N, Bock-Hensley O, Emmerich P, Günther S, Becker N, Niedrig M, Schmidt-Chanasit J. 2011. Medical importance of Sindbis virus in south-west Germany. Journal of Clinical Virology 52:278-279.

32. Kaboré DPA, Exbrayat A, Charriat F, Soma DD, Sawadogo SP, Ouédraogo GA, Tuaillon E, Van de Perre P, Baldet T, Morel C, Dabiré RK, Gil P, Gutierrez S. 2025. A metagenomics survey of viral diversity in mosquito vectors allows the first detection of Sindbis virus in Burkina Faso. PLoS One 20:e0323767. PMC12161561

33. Karabatsos N. 1985. International catalogue of arboviruses : including certain other viruses of vertebrates, 3rd ed. Published for the Subcommittee on Information Exchange of the American Committee on Arthropod-borne Viruses by the American Society of Tropical Medicine and Hygiene, San Antonio, Tex.

34. Kokernot RHH, Smithburn HM, Kausrud AD. 1956. A new arthropod-borne virus isolated in Uganda. 5:400-406.

35. Koren R, Bassal R, Shohat T, Cohen D, Mor O, Mendelson E, Lustig Y. 2019. Presence of Antibodies against Sindbis Virus in the Israeli Population: A Nationwide Cross-Sectional Study. Viruses 11. PMC6630228

36. Korhonen EM, Suvanto MT, Uusitalo R, Faolotto G, Smura T, Sane J, Vapalahti O, Huhtamo E. 2020. Sindbis Virus Strains of Divergent Origin Isolated from Humans and Mosquitoes During a Recent Outbreak in Finland. Vector Borne Zoonotic Dis 20:843-849. PMC7699012

37. Kozuch O, Labuda M, Nosek J. 1978. Isolation of sindbis virus from the frog Rana ridibunda. Acta Virol 22:78.

38. Kurkela S, Manni T, Myllynen J, Vaheri A, Vapalahti O. 2005. Clinical and laboratory manifestations of Sindbis virus infection: prospective study, Finland, 2002-2003. J Infect Dis 191:1820-9.

39. Liang G-D, Li L, Zhou G-L, Fu S-H, Li Q-P, Li F-S, He H-H, Jin Q, He Y, Chen B-Q, Hou Y-D. 2000. Isolation and complete nucleotide sequence of a Chinese Sindbis-like virus. The Journal of general virology 81:1347-51.

40. Ling J, Smura T, Lundstrom JO, Pettersson JH, Sironen T, Vapalahti O, Lundkvist A, Hesson JC. 2019. Introduction and Dispersal of Sindbis Virus from Central Africa to Europe. J Virol 93. PMC6675900

41. Lundström JO, Hesson JC, Schäfer ML, Östman Ö, Semmler T, Bekaert M, Weidmann M, Lundkvist Å, Pfeffer M. 2019. Sindbis virus polyarthritis outbreak signalled by virus prevalence in the mosquito vectors. PLoS Negl Trop Dis 13:e0007702. PMC6738656

42. Lundström JO, Lindström KM, Olsen B, Dufva R, Krakower DS. 2001. Prevalence of sindbis virus neutralizing antibodies among Swedish passerines indicates that thrushes are the main amplifying hosts. J Med Entomol 38:289-97.

43. Lundstrom JO, Pfeffer M. 2010. Phylogeographic structure and evolutionary history of Sindbis virus. Vector Borne Zoonotic Dis 10:889-907.

44. Lundström JO, Turell MJ, Niklasson B. 1993. Viremia in three orders of birds (Anseriformes, Galliformes and Passeriformes) inoculated with Ockelbo virus. Journal of wildlife diseases 29:189-195.

45. Lundstrom JO, Vene S, Espmark A, Engvall M, Niklasson B. 1991. Geographical and Temporal Distribution of Ockelbo Disease in Sweden. Epidemiology and Infection 106:567-574.

46. Lvov DK, Skvortsova TM, Berezina LK, Gromashevsky VL, Yakovlev BI, Gushchin BV, Aristova VA, Sidorova GA, Gushchina EL, Klimenko SM, et al. 1984. Isolation of Karelian fever agent from Aedes communis mosquitoes. Lancet 2:399-400.

47. Lwande OW, Naslund J, Lundmark E, Ahlm K, Ahlm C, Bucht G, Evander M. 2019. Experimental Infection and Transmission Competence of Sindbis Virus in Culex torrentium and Culex pipiens Mosquitoes from Northern Sweden. Vector Borne Zoonotic Dis 19:128-133. PMC6354595

48. M'Ghirbi Y, Mousson L, Moutailler S, Lecollinet S, Amaral R, Beck C, Aounallah H, Amara M, Chabchoub A, Rhim A, Failloux AB, Bouattour A. 2023. West Nile, Sindbis and Usutu Viruses: Evidence of Circulation in Mosquitoes and Horses in Tunisia. Pathogens 12. PMC10056592

49. Maar SA. 1980. A case of sindbis virus infection in Zimbabwe. Cent Afr J Med 26:161-2.

50. Malherbe H, Strickland-Cholmley M, Jackson AL. 1963. Sindbis virus infection in man. Report of a case with recovery of virus from skin lesions. S Afr Med J 37:547-52.

51. Matthew DA, Karlsson E, Izang JA, Isberg L, Näslund J, Sjödin A, Ottosson U, Lwande OW, Waldenström J. 2025. First detection of Sindbis virus in wild birds in Nigeria. Sci Rep 15:24621. PMC12241308

52. McIntosh BM, Jupp PG. 1979. Infections in sentinel pigeons by Sindbis and West Nile viruses in South Africa, with observations on Culex (Culex) univittatus (Diptera: Culicidae) attracted to these birds. J Med Entomol 16:234-9.

53. McIntosh BM, Jupp PG, Dos Santos I, Meenehan GM. 1976. Epidemics of West Nile and Sindbis viruses in South Africa with Culex (Culex) univittatus Theobald as vector. South African Journal of Science 72:295–300.

54. Meno K, Yah C, Mendes A, Venter M. 2021. Incidence of Sindbis Virus in Hospitalized Patients With Acute Fevers of Unknown Cause in South Africa, 2019-2020. Front Microbiol 12:798810. PMC8860305

55. Niklasson B, Espmark A, Leduc JW, Gargan TP, Ennis WA, Tesh RB, Main AJ. 1984. Association of a Sindbis-Like Virus with Ockelbo Disease in Sweden. American Journal of Tropical Medicine and Hygiene 33:1212-1217.

56. Nir Y, Avivi A, Lasovski Y, Margalit J, Goldwasser R. 1972. Arbovirus activity in Israel. Isr J Med Sci 8:1695-701.

57. Nir Y, Goldwasser R, Lasowski Y, Avivi A. 1967. Isolation of arboviruses from wild birds in Israel. Am J Epidemiol 86:372-8.

58. Norder H, Lundström JO, Kozuch O, Magnius LO. 1996. Genetic relatedness of Sindbis virus strains from Europe, Middle East, and Africa. Virology 222:440-5.

59. Omondi D, Masiga DK, Ajamma YU, Fielding BC, Njoroge L, Villinger J. 2015. Unraveling Host-Vector-Arbovirus Interactions by Two-Gene High Resolution Melting Mosquito Bloodmeal Analysis in a Kenyan Wildlife-Livestock Interface. PLoS One 10:e0134375. PMC4521840

60. Papapanagiotou J, Kyriazopoulou V, Antoniadis A, Batíková M, Gresíková M, Sekeyová M. 1974. Haemagglutination-inhibiting antibodies to arboviruses in a human population in Greece. Zentralbl Bakteriol Orig A 228:443-6.

61. Pavlatos M, Smith CE. 1964. ANTIBODIES TO ARTHROPOD-BORNE VIRUSES IN GREECE. Trans R Soc Trop Med Hyg 58:422-4.

62. Porterfield JS, Ash JS. 1966. Arbovirus antibodies in avian sera. Nature 212:431-2.

63. Renaudet J, Jan C, Ridet J, Adam C, Robin Y. 1978. [A serological survey of arboviruses in the human population of Senegal]. Bull Soc Pathol Exot Filiales 71:131-40.

64. Sane J, Kurkela S, Putkuri N, Huhtamo E, Vaheri A, Vapalahti O. 2012. Complete coding sequence and molecular epidemiological analysis of Sindbis virus isolates from mosquitoes and humans, Finland. J Gen Virol 93:1984-1990.

65. Shakya R, Tryland M, Vikse R, Romano JS, Asbakk K, Nymo IH, Mehl R, Evander M, Ahlm C, Vapalahti O, Lwande OW, Putkuri N, Johansen W, Soleng A, Edgar KS, Andreassen AK. 2022. Inkoo and Sindbis viruses in blood sucking insects, and a serological study for Inkoo virus in semi-domesticated Eurasian tundra reindeer in Norway. Virol J 19:99. PMC9166600

66. Sigei F, Nindo F, Mukunzi S, Ng'ang'a Z, Sang R. 2018. Evolutionary analyses of Sindbis virus strains isolated from mosquitoes in Kenya. Arch Virol 163:2465-2469.

67. Storm N, Weyer J, Markotter W, Kemp A, Leman PA, Dermaux-Msimang V, Nel LH, Paweska JT. 2014. Human cases of Sindbis fever in South Africa, 2006-2010. Epidemiol Infect 142:234-8. PMC9151170

68. Storm N, Weyer J, Markotter W, Leman PA, Kemp A, Nel LH, Paweska JT. 2013. Phylogeny of Sindbis virus isolates from South Africa. Southern African Journal of Epidemiology and Infection 28:207-214.

69. Streng K, Holicki CM, Hesson JC, Graham H, Chandler F, Krol L, Blom R, Münger E, van der Linden A, Koenraadt CJM, Schrama M, de Saint Lary CB, Visser LG, Munnink BO, Lundkvist Å, Koopmans MPG, van der Jeugd HP, van der Poel WHM, Sikkema RS. 2025. Local Circulation of Sindbis Virus in Wild Birds and Horses, the Netherlands, 2021-2022. Emerg Infect Dis 31:863-866. PMC11950264

70. Suvanto MT, Uusitalo R, Otte Im Kampe E, Vuorinen T, Kurkela S, Vapalahti O, Dub T, Huhtamo E, Korhonen EM. 2022. Sindbis virus outbreak and evidence for geographical expansion in Finland, 2021. Euro Surveill 27. PMC9358406

71. Taylor RM, Hurlbut HS, Work TH, Kingston JR, Frothingham TE. 1955. Sindbis virus: a newly recognized arthropodtransmitted virus. Am J Trop Med Hyg 4:844-62.

72. Tempera G, Guglielmino S, Pappalardo G, Castro A. 1980. Haemagglutination inhibition antibodies against Sindbis virus in human and bovine sera in Eastern Sicily. Acta Virol 24:157.

73. Tomori O, Fabiyi A. 1976. Antibodies against arboviruses in Sierra Leone. Trop Geogr Med 28:239-43.

74. Uryvaev LV, Vasilenko VA, Parasiuk NA, Ionova KS, Gushchina EA, Kullapere AA, Leĭbak E, L'Vov D K. 1992. [The isolation and identification of the Sindbis virus from migratory birds in Estonia]. Vopr Virusol 37:67-70.

75. Uusitalo R, Siljander M, Culverwell CL, Hendrickx G, Linden A, Dub T, Aalto J, Sane J, Marsboom C, Suvanto MT, Vajda A, Gregow H, Korhonen EM, Huhtamo E, Pellikka P, Vapalahti O. 2021. Predicting Spatial Patterns of Sindbis Virus (SINV) Infection Risk in Finland Using Vector, Host and Environmental Data. Int J Environ Res Public Health 18. PMC8296873

76. Vesenjak-Hirjan J, Punda-Polić V, Dobe M. 1991. Geographical distribution of arboviruses in Yugoslavia. J Hyg Epidemiol Microbiol Immunol 35:129-40.

77. Weinbren MP, Kokernot RH, Smithburn KC. 1956. Strains of Sindbis-like virus isolated from culicine mosquitoes in the Union of South Africa. I. Isolation and properties. S Afr Med J 30:631-6.

78. Wills WM, Jakob WL, Francy DB, Oertley RE, Anani E, Calisher CH, Monath TP. 1985. Sindbis virus isolations from Saudi Arabian mosquitoes. Trans R Soc Trop Med Hyg 79:63-6.

79. Woodall J, Williams M, Ellice J. 1962. Sindbis infection in man. East Afr Virus Res Inst Rep 12:17.

80. Woodall JP, Williams MC, Corbet PS, Haddow AJ. 1964. THE ISOLATION OF SINDBIS VIRUS FROM THE MOSQUITO MANSONIA (COQUILLETTIDIA) FUSCOPENNATA (THEOBALD) IN UGANDA. Ann Trop Med Parasitol 58:383-9.

81. Ziegler U, Fischer D, Eiden M, Reuschel M, Rinder M, Müller K, Schwehn R, Schmidt V, Groschup MH, Keller M. 2019. Sindbis virus- a wild bird associated zoonotic arbovirus circulates in Germany. Veterinary Microbiology 239:108453.
